# The Market Dynamics of Generic Medicines in the Private Sector of 19 Low and Middle Income Countries between 2001 and 2011: A Descriptive Time Series Analysis

**DOI:** 10.1371/journal.pone.0074399

**Published:** 2013-09-30

**Authors:** Warren A. Kaplan, Veronika J. Wirtz, Peter Stephens

**Affiliations:** 1 Center for Global Health and Development, Boston University School of Public Health, Boston, Massachusetts, United States of America; 2 World Health Organization Collaborating Centre for Pharmaceutical Policy, Boston University, Boston, Massachusetts, United States of America; 3 IMS Health, London, United Kingdom; 4 World Health Organization Collaborating Centre for Pharmacoepidemiology and Pharmaceutical Policy Analysis, Utrecht Institute for Pharmaceutical Sciences (UIPS), Utrecht, The Netherlands; York University, Canada

## Abstract

This observational study investigates the private sector, retail pharmaceutical market of 19 low and middle income countries (LMICs) in Latin America, Asia and the Middle East/South Africa analyzing the relationships between volume market share of generic and originator medicines over a time series from 2001 to 2011. Over 5000 individual pharmaceutical substances were divided into generic (unbranded generic, branded generic medicines) and originator categories for each country, including the United States as a comparator. In 9 selected LMICs, the market share of those originator substances with the largest decrease over time was compared to the market share of their counterpart generic versions. Generic medicines (branded generic plus unbranded generic) represent between 70 and 80% of market share in the private sector of these LMICs which exceeds that of most European countries. Branded generic medicine market share is higher than that of unbranded generics in all three regions and this is in contrast to the U.S. Although switching from an originator to its generic counterpart can save money, this narrative in reality is complex at the level of individual medicines. In some countries, the market behavior of some originator medicines that showed the most temporal decrease, showed switching to their generic counterpart. In other countries such as in the Middle East/South Africa and Asia, the loss of these originators was not accompanied by any change at all in market share of the equivalent generic version. For those countries with a significant increase in generic medicines market share and/or with evidence of comprehensive “switching” to generic versions, notably in Latin America, it would be worthwhile to establish cause-effect relationships between pharmaceutical policies and uptake of generic medicines. The absence of change in the generic medicines market share in other countries suggests that, at a minimum, generic medicines have not been strongly promoted.

## Introduction

In recent years, the growth of government health programmes, coupled with major and disruptive shortfalls in financing, have forced governments to realize that the provision of low-cost, quality assured medicines will need to take on increasing importance [1]–[2]. To lower total pharmaceutical expenditures, many high income countries have implemented a series of policies to promote the use of generic medicines [3]. In Europe, for example, generic medicines volume share (the European data refers to the unprotected market of pharmaceuticals which includes only those products that are have never been, or are no longer, protected by patents) increased from 42% in 2005 to 49.0% in 2009 [4]. With respect to individual countries, increases in the market share of generic medicines have been documented in Germany, France and Sweden between 2006 and 2009 [4]–[5]. In absolute terms, in 2009 generic medicines were 65% of the total market by volume in Germany, 60% in the UK, 40% in France and 30% in Spain and Italy [4].

The United States has also implemented policies to promote the use of generic medicines, most notably the Drug Price Competition and Patent Term Restoration Act, informally known as the “Hatch-Waxman Act” [6]. Between 1984 and 2005, generic medicines in the U.S. increased from 19% to 54% of the total pharmaceutical market volume [7] and in the last decade, of all U.S. prescriptions dispensed in retail pharmacies, 80% by volume were filled using generic medicines [8]. Strong support from Medicaid and private health insurances to contain costs as well as from state laws requiring generic substitution [7] has been identified as the main factors for this increase.

Apart from these high income countries, many low and middle income countries (LMICs) have introduced policies to promote uptake of generic medicines (e.g. South Africa, Brazil, Philippines) [9]. Their impact could be substantial [10]–[11] but we know far less about the effect of pro-generic medicine policies in LMICs than in high income countries [9]. Indeed, we know comparatively little about the private sector pharmaceutical market in low and middle income countries (LMICs) as compared to the public sector LMIC pharmaceutical markets [12]–[14] and even less about the market dynamics between originator/brand name and generic versions of the same medicine.

In this observational, retrospective study, we provide data that answers the following questions: What are the trends of originator and generic medicines market share in the private sector of selected LMICs over the last 10 years? What patterns can we observe in the relationship between the market share of an originator and its generic medicine counterpart in the private sector of LMICs? We also suggest some potential drivers of these market relationships.

## Materials and Methods

### Data sources

We obtained retail private sector sales data (prescription and over-the-counter (OTC)) from IMS Health (www.imshealth.com) on the aggregated volume of oral (including oral liquids) pharmaceutical products, excluding contraceptives, herbal medicines, vitamins, insulins and neurotonics for 19 LMICs and the United States from 2001 to 2011. The raw data is available upon request by third party researchers for non-commercial purposes at the approval of the IMS Health Global Health Research Program. [15] The LMICs (as defined by the World Bank [16]) are from three different geographical regions: Latin America and the Caribbean (“LAC”: Argentina, Brazil, Chile, Colombia, Dominican Republic, Ecuador, Mexico, Peru, Uruguay, Venezuela); Middle East plus South Africa (MeSA) (Egypt, Jordan, Morocco, Tunisia, South Africa) and Asia (Bangladesh, Pakistan, Philippines, Thailand). The selection of the LMIC was guided by the availability of data from the retail sector in the three geographical regions. Even though this is not a representative sample of countries in each region, the countries chosen are important pharmaceutical markets in terms of their value in the respective regions. We used data from the United States as comparator. In this regard, the US market has the most well-understood dynamics of the countries in our study and in 2011 it had about 34% of global pharmaceutical spending. It is the largest pharmaceutical market in the world and one of the largest of generic medicines markets. Per capita spending on pharmaceuticals (2005 dollars) in the US was 5 times that of Brazil (the largest market in the LAC). [17].

In the LMICs under review here, the data primarily reflect the private sectors that receive out-of-pocket payments although in some countries the private sector also includes the private insurance sector and governmental social security. Significantly, volume data represent either purchase or dispensing by the supply chain, rather than actual consumption by patients.

We excluded contraceptives, insulin, herbals, neurotonics and vitamins because the category includes many molecules that are not considered to be new active substances and therefore do not have an “originator” under our classification system (See next section).

The retail sales volume of oral solids and oral liquids was reported in “standard units” (SU). For oral solids one SU is one tablet or capsule. For oral liquids, one SU is 5ml. Our analysis focuses on market share expressed as percentage of retail market volume. The Defined Daily Dose (DDD) which is the standard method when studying medicines utilization was not used as, converting SU into DDD for the substantial number of combination products is difficult. In interpreting the volume trends described below, it should be borne in mind that the exact same set of pharmaceutical products is not being compared. The range of products distributed in the private sector differs by country and has differed over time. What we are measuring are the various volume components of the private pharmaceutical market as a percentage of the total private pharmaceutical market volume.

Data on the private sector sales volume is country-specific and collected from various stages in the retail pharmaceutical supply chain (i.e. from pharmaceutical manufacturers and importers, wholesalers, distributors, and sub-distributors of medicines) depending on the country.

### Data analysis

For each country, the database is populated with aggregated annual sales volumes coded for the following five categories of products: originator brands, licensed brands, “Other” brands, unbranded products and “Patent Not Applicable” product categories. We regrouped those five categories into four and renamed them (“other brands” as “branded generics” for consistency with the literature [18]):

1. **Originator products:** “Originator” products are those products first authorized in a given country for marketing (normally as a patented product) on the basis of the documentation of its efficacy, safety, and quality, according to requirements at the time of authorization. Originator products that are marketed by a company under the terms of a licensing agreement with the originator are defined as “Licensed Brands”. These two particular categories were combined for the present analysis and are combined and named hereafter as “originator” medicines.
2. **Unbranded generic products:** Non-originator products sold under an international non-proprietary name (INN) (i.e., the generic name of the ingredient molecule(s)) rather than a brand name. That is, they are products that are off-patent without a trade name and from a single source or co-licensed.
3. **Branded generic products:** Branded generics are non-originator products. They can be either novel dosage forms of off-patent products produced by a manufacturer that is not the originator of the molecule, or a molecule copy of an off-patent product with a trade name produced by a manufacturer not the originator. In other words, products sold under brand names by a company NOT the originator company and for which there is no evidence of a licensing agreement between them fall into this category.
4. **Patent N/A products:** These are products whose patent status could not be, or has not been, defined under the IMS classification with any certainty and thus could not be placed into any of the other three categories. Because of this uncertainty, we did not use this category in our subsequent analysis of the market share. This introduced some limitations as discussed below.

We converted the standard unit volume of medicines for three categories (see above) into their respective percentages to obtain outcome measurements, as follows:

- **(i)“Total generic market share”:** the percentage of total annual private sector sales volume of branded generic medicines plus unbranded generic medicines divided by the total annual medicines private sector sales volume (originator plus licensed plus branded generic plus unbranded generic medicines).

Total generic market share  =  (unbranded + branded generic medicines)/(unbranded + branded generic + originator + licensed medicines).

- **“Branded generic medicines market share”:** the percentage of annual private sector sales volume of branded generic medicines divided by total medicines private sector sales volume, as defined immediately above.
- **“Unbranded generic medicines market share”:** the percentage of annual private sector sales volume of unbranded generic medicines divided by total medicines private sector sales volume, as defined above.

We took as the “regional” market share the **median** value of the respective market shares for all countries in a given region (LAC, Asia, MeSA) of the different categories (unbranded, branded generic, originator) in a given year. Thus, for the LAC region, the median regional branded generic market share is the median value of the branded generic market share for the 10 different LAC countries. For the metric “total generic market share” for the LAC, we calculated the median LAC market share for each individual category of generic (as described above) and summed median regional values of branded + unbranded markets.

### Quantifying the volume relationships between ‘originator’ and ‘generic’ medicines

We tested whether a decrease in percent market share of an originator products and any concomitant increase in market share of the counterpart generic products (branded + unbranded generic versions) can be explained as an intentional “switch” of the same pharmaceutical substance from originator to generic. We chose those countries for which there was at least an overall 6% decrease in percentage market share of all originator products between 2001 and 2011: these countries being South Africa, Colombia, Brazil, Philippines, Peru, Ecuador, Venezuela, Mexico and Jordan. We used the United States as a comparator.

By looking at specific pharmaceutical substances per group (originator, branded generic, unbranded generic) we were able to determine if the decrease in market share of a specific originator pharmaceutical substance was accompanied by an increase in its counterpart unbranded and/or branded generic market share (s).

We used the disaggregated data on yearly volumes of a total 5131 different pharmaceutical substances (molecules or combinations of molecules) for the 10 countries listed above for all years from 2001 to 2011. For each country, we calculated the difference in volume market share (as a % of the total volume of all pharmaceuticals for all categories (exclusive of the “Patent N/A” category) between 2001 and 2011. For originator pharmaceutical substances, we ranked them by this so-called ”delta originator” with the largest negative delta first, and selected for further analysis the top ranked 30 in this list (hereafter called the “top 30 list”). For each of these top 30 originator pharmaceutical substances, we compared its loss in market share with the change in market share (delta 2001–2011) of the exact counterpart unbranded and branded pharmaceutical substances (“delta unbranded” and “delta branded generic”, respectively).

For each specific ‘originator’ pharmaceutical substance in the top 30 list for each country we calculated a simple diagnostic ratio: ((delta unbranded+ delta branded generic)/ delta originator)) to detect whether there was a net growth, loss or no net change in market share for the generic counterparts to each these top 30 pharmaceutical substance between 2001 and 2011. The magnitude of the diagnostic provides quantitative information about the relative magnitude of the respective change in market shares. See Table 1.

**Table 1:** “Diagnostic ratios”: definitions, examples and explanation.

Delta (unbranded + branded generic)	Delta (Originator)	Delta (unbranded + branded generics/delta (Originator)	Examples	Explanation of changes in market share
Positive = net gain in generics	Always negative	Positive	Ratio greater than zero but <1 e.g., Ratio = +0.5	Generic growth half that of Originator loss (Category B)
		(We divided this by the corresponding delta (originator) which is always a negative number and took the absolute value to yield a positive diagnostic	Ratio = 1	Generic growth matched by originator loss
			Ratio = >1, e.g., 3.5	Generic growth 3.5 times that of originator loss (Category A)
Negative = net loss in generics	Always negative	Negative We divided this by the corresponding delta (originator) and multiplied the fraction by minus 1 to yield a negative diagnostic.)	Ratio less than zero, e.g. –0.5	Loss of generic market share twice that of originator loss (Category C)
Zero = no generic on market at any time		Zero		Category D

### Inferences about patent protection

For Brazil and the U.S., we had information on whether or not the top 30 pharmaceutical substances were under patent during the relevant time period 2001–2011. We did not have this information for the other countries. Instead, we developed some inferences about the presence of patent protection by checking if the originator substances in the top 30 list for all other LMICs besides Brazil had a generic counterpart in Q4 2000. If so, this would suggest that the originator patents were either ignored or non-existent for these products over the subsequent period 2001–2011. Conversely, we looked for “ top 30 ” originator substances with no generic product marketed at the end of 2000 but for which there was a subsequent diagnostic ratio for 2001–2011 greater than 1 (i.e., subsequent rapid growth of generic market share greater than the decrease in originator market share). This would be a strong inference of rapid generic “replacement” of an originator.

### Sensitivity analysis

As the retail data used for this study is based on audits from the distribution chain, it is almost inevitable that the number of outlets/entities in this chain would change over time and possibly impact the volume data. Such changes can be primarily due to inclusion of generic products from new companies, incorporation of sales of private label products that belong to pharmacy chains, the additional new data suppliers and new wholesalers into the audit. Reclassification of products according to official lists would not change the data sources but may possibly rates of generic uptake. Hence IMS routinely performs a validation of the retail sales data by comparing estimated yearly sales volumes for each product pack with the manufacturer's estimated or provided sales volumes supplied to the retail sector. For the countries under study here, the largest variation registered for the study period was for Jordan in which the manufacturer estimated total sales volume for all medicines categories over all years to be, on average, 22% percent more than what the audits recorded (data not shown here). For Brazil the manufacturer estimated the actual total sales volume on average 5% higher. We chose Jordan because this apparent bias is the largest among the lower income countries and Brazil this is the largest bias for upper middle countries.

Although the ‘bias’ in this estimation probably varies between our categories, it is reasonable to assert that the manufacturers that supply data for validation are those that use the IMS data. In general this will tend to include a higher proportion of branded, larger manufacturers than unbranded. Our validation is thus more likely to be most representative of larger companies and less representative of the smaller companies. To estimate the possible impact of such bias on market share, we did a sensitivity analysis for Jordan in which we assumed that the volume of unbranded generics was actually 22% higher each year than reported. We recalculated the unbranded generic market share and computed the difference in market share with and without unbranded generic ‘bias’. We did the same analysis for unbranded generics in Brazil assuming the volume was actually 5% higher each year.

## Results

### I. Regional Market Share:

#### A. Total generic medicines market share

**Figure 1:**
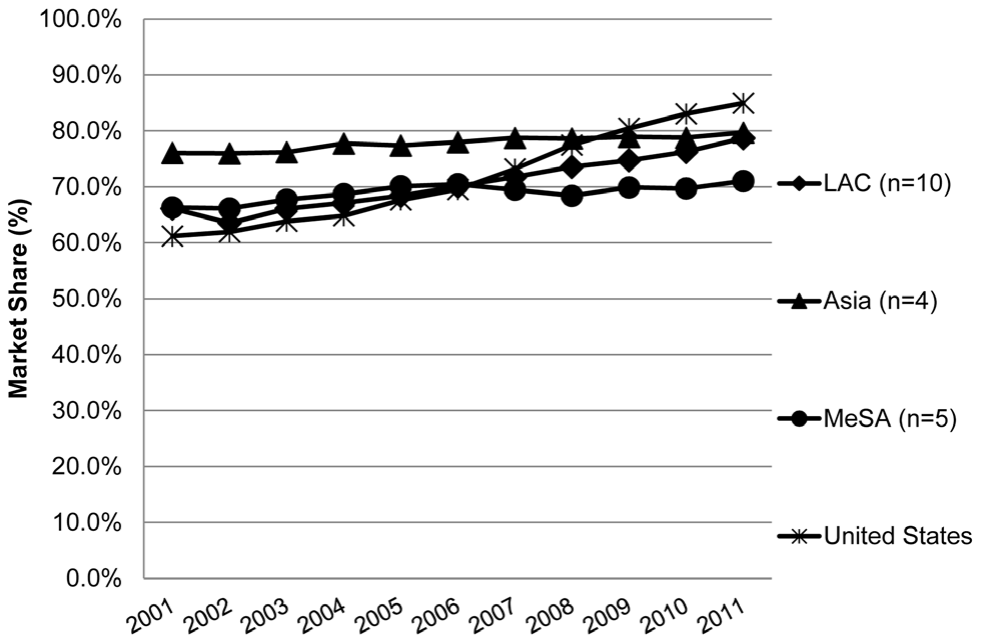
Time series of “total generic market share” in 19 LMICs and the U.S. LEGEND: The trend (change in total generic market share/yr) was calculated using a simple linear regression model. Trend: United States 1.54%/yr; LAC 1.12%/yr; Middle East plus South Africa (MeSA) 0.38%/yr; Asia 0.31%/yr. A t test for regressions were all significant [p<0.05].

**Figure 2:**
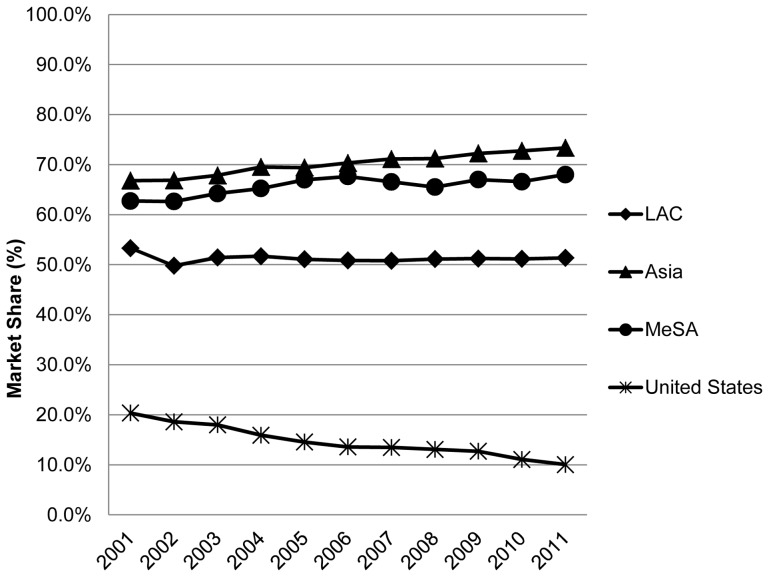
Time series “branded generic” market share in 19 LMICs and the U.S. LEGEND: The trend (change in branded generic market share/yr) was calculated using a simple linear regression model.Trend: United States −1.15%/yr; LAC −0.34%/yr; Middle East plus South Africa (MeSA) 0.47%/yr; Asia 0.61%/yr. A t test for regressions were all significant [p<0.05]. The number of countries in Figure 2 are the same as in Figure 1.

**Figure 3:**
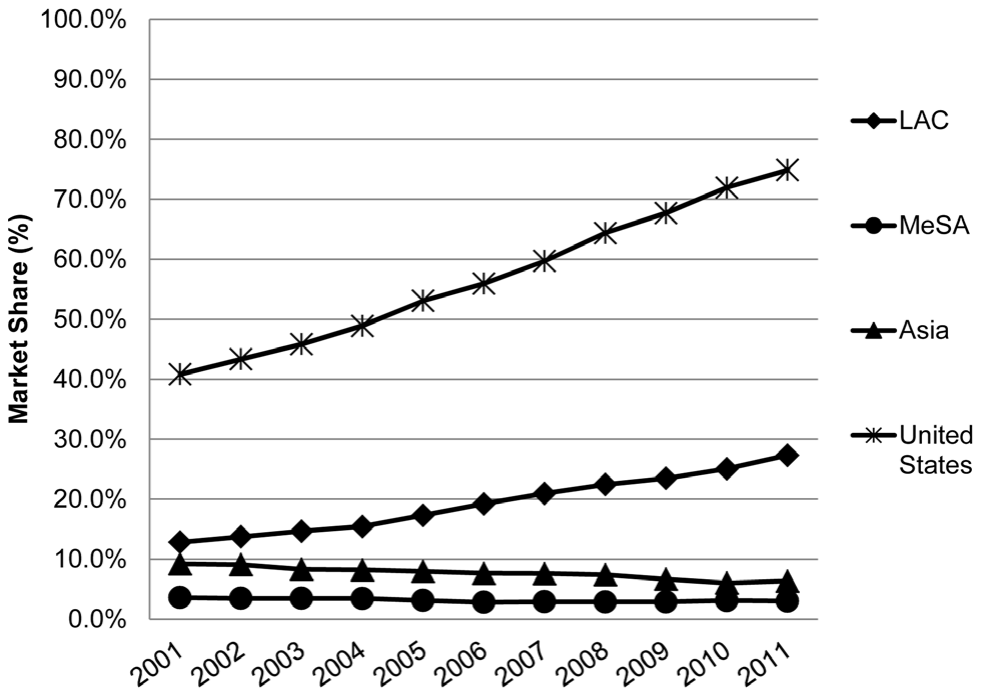
Time series of “unbranded generic” market share in 19 LMICs and the U.S. LEGEND: The trend (change in branded generic market share/yr) was calculated using a simple linear regression model.Trend: United States 2.90%/yr; LAC 1.46%/yr; Middle East plus South Africa (MeSA) −0.08%/yr; Asia −0.27%/yr. A t test for regressions were all significant [p<0.05]. The number of countries in Figure 3 are the same as in Figure 1.

The temporal changes in total generic market, as defined above, are in Figure 1 (where n =  number of countries in the region). Each point in the time series for a given region is the median value of the individual countries in that region (See also Figs 2–3, where “n” is the same as in Fig 1).

The share of the market volume of generic medicines (unbranded plus branded, excluding originator) in the LAC region increased from 66% to 78% during this 10-year period. These increases are about three times that of the Middle East plus SA (MeSA) and Asia countries studied. Although the Asian countries studied here had the highest absolute total generic market share over this time period (>70%), they showed the smallest change over time. The United States market volume share of generic medicines shows the highest increase in comparison to the three regions studied (growth from 61% to 85%).

#### B. Branded generic medicines

The median market fraction of branded generics medicines in all regions is greater than 50% meaning that the majority of ‘generic’ medicines in the private sector of all 19 LMICs are branded medicines whose manufacture is not licensed by the maker of the corresponding originator product (Figure 2). This is in sharp contrast to the US where less than 20% of the market share corresponds to this category of medicines and where this value decreased over time. Whereas the median volume share of branded generics in the MeSA and Asian countries increased during the study period, for the LAC region it slightly decreased over time but the LAC region is consistently the lowest compared to the MeSA and Asian countries.

#### C. Unbranded generic medicines

The median market fraction for unbranded generic medicines in the LAC countries studied here (although much lower than the U.S. in absolute terms) more than doubled from 13% to 27% 2001–2011at a rate of 1.46%/yr (Figure 1). In contrast, the volume share of unbranded generics slowly decreased in the Asian countries in the study period (from about 8% to 6%: rate of −0.27%/yr) and was very low and substantially unchanged in countries of the MeSA countries under study (3.6% to 2.9%: rate of −0.08%/yr). In contrast, the unbranded generic medicines volume in the United States over this same time period increased from 41% to 68% of the total market (at a rate of 2.90%/yr), with the highest market share and positive trend as compared to the three regions.

### II. Changes in Private Sector Market share in individual countries

With respect to individual countries, twelve of them showed aggregate increases in percent market share of unbranded generics between 2001 and 2011 (“delta” range: 0.3% to 22.3%) (Table 2). Eight of these twelve countries were in the LAC region. Thirteen countries showed aggregate increases in percent market share of branded generics (“delta” range: 2.8% to 26.7), with three of the five countries from the Asian region (Philippines, Pakistan and Bangladesh). Six countries showed aggregate increases in both unbranded and branded generics (Mexico, Argentina, South Africa, Jordan, Morocco, Philippines). In 2011, the countries with the highest share of private sector originator medicines were Tunisia (37.2%), Pakistan (35.6%), Mexico (34.8%), and Morocco (31.9%). Nonetheless, the market share for the entire originator market decreased in all countries, with large decreases in certain countries in Latin America (e.g., Brazil, Mexico, Colombia, Peru, Venezuela, Ecuador, Uruguay) as well as Jordan and South Africa These countries showed a more than 6% market share decrease in total originator market between 2001 and 2011 (Table 2).

**Table 2:** Market share of unbranded, “other” (branded generic), and originator products in 2001 and 2011 by country.

	Unbranded generics market share % (2001)	Unbranded generics market share % (2011)	*Delta		Branded Generic share % (2001)	Branded Generic share % (2011)	*Delta		**Originator share % (2001)	**Originator share % (2011)	*Delta
Brazil	8.9	31.2	22.30%	Argentina	55.7	82.4	26.70%	Thailand	7.2	7	−0.20%
Colombia	38	57.7	19.70%	Mexico	41.5	51.2	9.70%	Argentina	29.5	29.1	−0.40%
Peru	19.6	38.6	19.00%	Philippines	61.7	70	8.30%	Pakistan	36.6	35.6	−1.00%
Venezuela	15	33	18.00%	Pakistan	53.2	60.3	7.10%	Chile	10	8.5	−1.50%
Mexico	1.2	14.1	12.90%	Bangladesh	86.6	92.2	5.60%	Dominican Republic	15.4	12.7	−2.70%
Ecuador	11.4	23.5	12.10%	South Africa	62.8	68.3	5.50%	Egypt	31.5	28.5	−3.00%
Uruguay	5.8	11.5	5.70%	Jordan	62.7	68	5.30%	Tunisia	40.9	37.2	−−3.70%
Argentina	7.1	11.4	4.30%	Thailand	71.9	76.8	4.90%	Bangladesh	10.6	6.9	−3.70%
South Africa	5.5	8.9	3.40%	Morocco	60.8	65.2	4.40%	Morocco	37.5	31.9	−5.60%
Jordan	4.6	6.3	1.70%	Chile	42.5	46.8	4.30%	Uruguay	19.3	13.7	−5.60%
Morocco	1.8	2.9	1.10%	Tunisia	55.5	59.8	4.30%	Ecuador	31.5	25	−6.50%
Philippines	8.2	8.5	0.30%	Egypt	65.3	68.5	3.20%	Jordan	32.7	25.7	−7.00%
Egypt	3.1	3	−0.10%	Dominican Republic	70.4	73.2	2.80%	Venezuela	35.4	28	−7.40%
Dominican Republic	14.2	14.1	−0.10%	Uruguay	74.9	74.8	−0.10%	Philippines	30	21.5	−8.50%
Tunisia	3.6	3	−0.60%	Brazil	53.3	51.6	−1.70%	South Africa	31.7	22.8	−8.90%
Bangladesh	2.8	1	−1.80%	Ecuador	57	51.5	−5.50%	Peru	20.7	11.5	−9.20%
Chile	47.5	44.7	−2.80%	Peru	53.30%	47.60%	−5.70%	Colombia	23.50%	10.30%	−13.20%
Thailand	20.9	16.2	−4.70%	Colombia	38.60%	32.00%	−6.60%	Mexico	54.20%	34.80%	−19.40%
Pakistan	10.2	4.20%	−6.00%	Venezuela	49.30%	39.00%	−10.30%	Brazil	37.80%	17.30%	−20.50%

Legend: *Delta  =  Difference between 2011 and 2001; **Originator market  =  includes licensed brands, as defined above.

### III. Market dynamics of originator and generic versions of individual pharmaceutical substances

We calculated the ‘diagnostic ratio’ previously described to test whether the decrease in a given originator market share was matched by an increase in market share of its counterpart generic version (branded and unbranded). In the 9 LMICs we selected for this analysis (Jordan, South Africa, Brazil, Mexico, Colombia, Peru, Venezuela, Ecuador, Uruguay), the top 30 originator pharmaceutical substances with the highest market share losses accounted for between 50 and 75% of the total loss of originator market share between 2001 and 2011.

**Figure 4:**
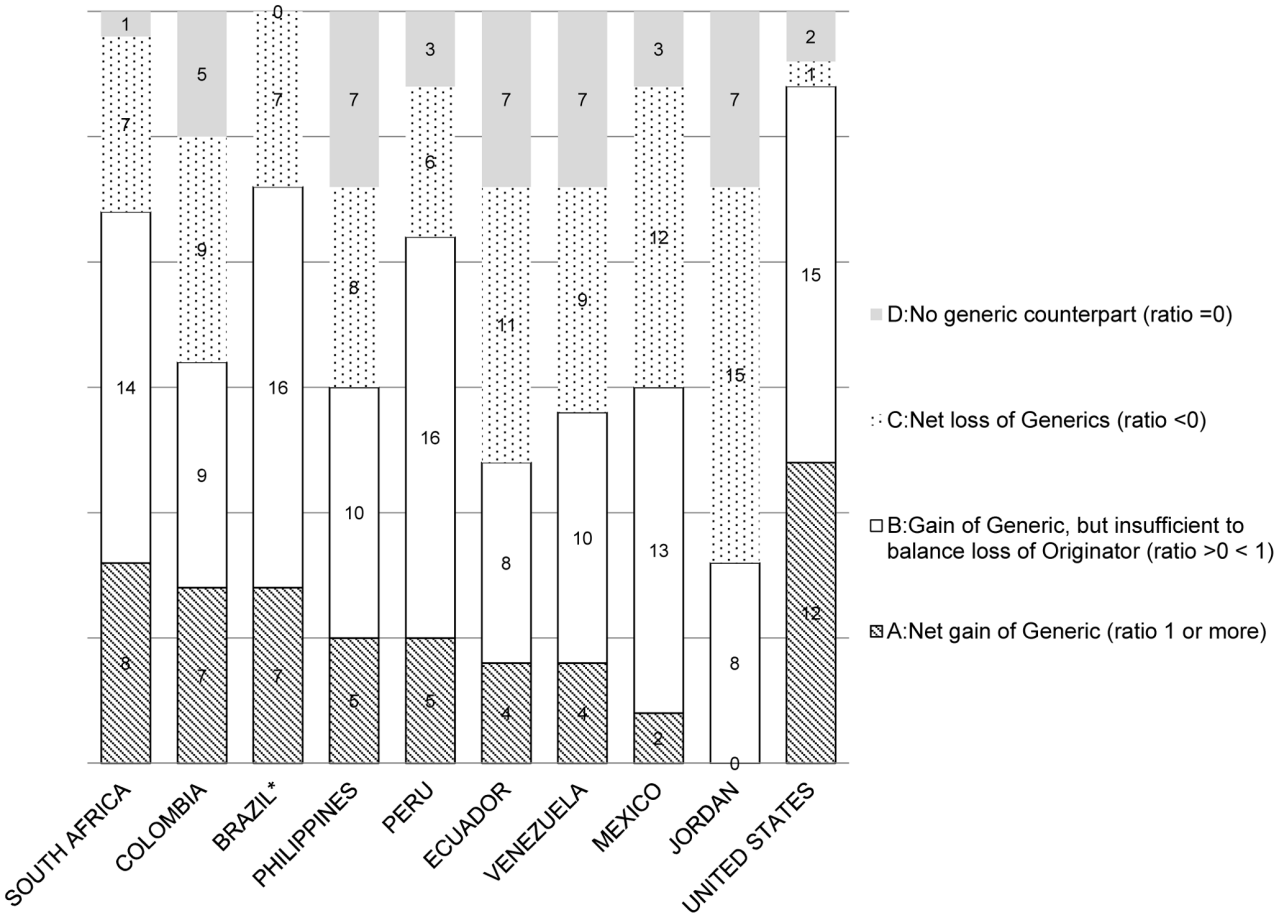
The distribution of diagnostic ratios (in the 4 categories) for each countries' 30 originator pharmaceutical substances. The number in each bar is the number of medicines falling into the respective category.

Figure 4 shows the distribution of the diagnostic ratios (in the 4 categories) for each countries' 30 originator pharmaceutical substances, including the United States. The number in each bar is the number of medicines falling into the respective category. In category A (“net generic gain”), the diagnostic ratio is 1 or more. Of the nine LMICs selected for analysis, South Africa displays the largest number of top 30 pharmaceutical substances in which the increase in generic market share of the substance was larger than the corresponding decrease in originator market share. Of all countries analyzed, the United States has the largest number of these category A pharmaceutical substances (12/30) and the largest total number of top 30 pharmaceutical substances (27/30) with a loss of originator and at least some corresponding increase in generic market share, i.e., sum of categories A and B. Brazil (23/30) and South Africa (22/30) are the LMICs with the largest number of category A and B pharmaceutical substances. Jordan was the only country of these nine LMICs which showed no generic replacement of any of the top 30 originator pharmaceutical substances over the study period (no “Category A” medicines). Indeed, for half of the top 30 originator substances on the Jordanian market between 2001 and 2011, there was also a loss of counterpart generic market share (15 “Category C” medicines).

In most countries, some of the “top 30” originator substances that lost market share did not have a generic counterpart on the market at all during 2001–2011 (“Category D”). These category “D” substances are listed in Table 3. The only exception was Brazil, in which all the top 30 originator substances had a generic counterpart on the market during 2001–2011 (no “Category D” medicines) (Figure 4).

**Table 3:** Pharmaceutical substances with decreasing originator market share (2001–2011) and no generic counterpart on market.

PERU	COLOMBIA	ECUADOR	URUGUAY	PHILIPPINES	VENEZUELA	SOUTH AFRICA	MEXICO	JORDAN	UNITED STATES
Acetylsalicylic acid/ chlorphanamine/ pseudoephedrine	Caffeine/diphen -hydramine/ ergotamine	Chlorphenamine/ salicylic acid	Diphenhydra- mine/guaifenesin	Betamethasone/ dexchlorphenir-amine	Betamethasone /dexchlorphenir-amine	Rofecoxib	Pseudo-ephedrine/ triprolidine	Amitriptyline	Celecoxib
Dextromethorphan/ diphenhydramine	Fluphenazine/ nortriptylline	Diphenhydramine	Diphenhydra-mine/ paracetamol/ phenylpropanol-amine	Betamethasone	Rofecoxib		Acetyl-salicylic acid/ Chlorphen-amine/ Pseudoephe-drine	Biperiden	Rofecoxib
	Metildigoxin	Dextromethorphan/ diphenhydramine		Betamethasone/ chlorphenamine	Celecoxib		Dexbrom-pheniramine/ pseudephed-rine	Clobutinol/ orciprenaline	
	Bromelains/dihydrocholic acid/ dimeticone/ metoclopramide/ pancreatin	Chlorphenamine/ phenylephrine/salicylamide		Diphenhydra-mine/guaifenesin	Ephedrine/ hydroxyzine/ theophylline			Clonazepam	
	Hydrochlorthia- zide/propranolol	Acetylsalicylic acid/caffeine/		Atropine diphenoxylate	Lorazepam			Flupentixol	
		Rofecoxib		Guaifenesin/ terbutaline	Metamizole sodium/ scopalamine butyl hydroxide			Clopamide/ dihydro- ertocristine/ reserpine	
		Calcium/copper/ dexpanthenol/ iron/magnesium/ manganese/ molybdenum/ multivitamins/ phosphorus/ potassium/ vitamin E/zinc		Ketotifen	Triazolam			Hydroxyzine	

Legend: Ingredients separated by backslash (/) are part of the same combination.

Some of the “top 30” originator molecules were commonly found in several countries, e.g., glibenclamide (antidiabetic), diclofenac (anti-inflammatory), sulfamethoxazole plus trimethoprim (antibiotic), amoxicillin (antibiotic) and alprazolam (psycoleptic) were common in eight countries (for more detailed description for the common molecules see Annex S1). For some of these above-identified molecules, the increase in generic market share was larger than the corresponding decrease in market share of the counterpart originator indicating an originator-to-generic switch (e.g., glibenclamide in Venezuela, diclofenac in Colombia, Uruguay and South Africa, amoxicillin in Colombia).

We can make some inferences about the presence of patent protection. Most of the originator products in the top 30 list for all LMICs had a generic counterpart in Q4 2000, suggesting that originator patents were either non-existent or perhaps ignored for these products over the period 2001–2011. In Brazil, we know that all the top 30 originator products lacked patent protection during 2001–2011 (data not presented here). However, we did observe that in other countries, for several substances there was a top 30 originator with no generic product marketed at the end of 2000 but for which there was a subsequent diagnostic ratio for 2001–2011 greater than 1 (i.e., subsequent rapid growth of generic market share greater than the decrease in originator market share): orlistat-Colombia; cyproheptadine-Ecuador; cefaclor and trimetazidine-Philippines; glibenclamide- Venezuela; loratidine; citalopram, meloxicam, omeprazole, simvastatin – all South Africa.

### IV. Sensitivity Analyses

For Brazil and Jordan, we assumed that, each year, the volume of unbranded generics was, respectively, 5% and 25% more than the audited volume. We calculated the potential error induced in the market share for these assumptions and for Brazil, the error is small (range: 0.28–1.1% underestimation of unbranded generic market share). For Jordan, the potential error induced is also fairly small (range: 0.71–1.02% underestimation of unbranded generic market share).

## Discussion

To our knowledge, this is the first such longitudinal analysis of the private sector generic medicine market in a large number of LMICs. We wish to bring out several points.

### 1. Total generic medicines market share in some LMICs exceeds that of many European countries

In 2001 the volume market share of generic medicines (unbranded + branded) was over 65% in all three regions which means that the 19 LMIC studied generally initially (in 2001) had a higher percentage of generic medicines market share than the United States and many European countries [5]. (See Figure 1, page 878). However, there has been little temporal change in market share of generic medicines (unbranded + branded) in at least half of the 19 LMIC studied, specifically in Asia and countries in the MeSA (Figure 1 present paper). This is in contrast to many European countries and the United States [5], where the generic medicines market share over this time period has increased at least 25 percent. See also Figures 1 and 3 of this present paper. Our results are in line with others describing similar increasing trends in the utilization of generic medicines in the US and Europe [19], [20].

### 2. Dominance of branded generics over unbranded generics as a class

In sharp contrast to the United States where the overwhelming majority of generic products are unbranded (Figure 3), branded generics by class are by far the dominant form of generic medicine in our private sector LMIC dataset (Figure 4). The countries in the MeSA we analyzed had by far the greatest preponderance of branded versus unbranded generics as a volume ratio (trend 17:1 to 23:1), followed by the Asian countries (trend 7 to10:1) and the LAC countries (trend 5:1 to 2:1) (data not presented here).

From a business perspective, launching a “branded” generic product may be a good choice in certain middle income countries where the ‘brand’ provides some perceived signal of assured quality over time. For instance, almost all medicines in India are sold under a trade/brand name and not an unbranded (INN) name [21]. Generic manufacturers aim to establish themselves in a particular product market by creating brand awareness, and, potentially, brand loyalty among prescribers and/or patients.

The promotion and marketing of branded generics by all these entities raises the question as to whether branded generics are likely to be more expensive than their unbranded counterparts. In Peru, for two ACE inhibitors (captopril, enalapril), three anti-ulcerants (lansoparzole, omeprazole, ranitidine) and two anti-diabetic agents (glibenclamide, metformin), the branded generic ranged from 26% more expensive (metformin) to 900% more expensive (enalapril) than its unbranded generic counterpart [22]. A study from Brazil found that unbranded products were more expensive than branded ones, the explanation being that unbranded products have to prove bioequivalence, and this cost is added to the consumer price [1]. There appears, however, to be little data in the literature on this type of price comparison between branded and unbranded generic medicines.

### 3. Some evidence for originator to generic “switching” exists for certain medicines in these markets

The cost savings of increased use of generic medicines can be substantial in LMICs [11]. Potentially, it is possible to improve cost-effective medicine use in the private sector if originator brands were to be switched to the lowest-priced generic equivalents available at medicine outlets [11]. The amount of saving would depend on the price difference between originator and generic equivalent. However, as our data suggest when disaggregated into individual pharmaceutical substances, the actual situation appears more complex than simple “switching”. One should not assume that, if market share of an originator has decreased, then its counterpart generic has increased. For many countries, this assumption does not hold.

In those nine LMICs whose private sector market shares we have disaggregated into their ‘diagnostic ratios” (Table 1), there appears to be a spectrum of market behaviors with respect to those originator medicines that lost market share, ranging from e.g., loss of originator market share without any generic counterpart on the market at all (Table 3, Figure 4: “Category D”) to a growth of counterpart generic volume share sufficient to overcome the decrease in originator volume share (Figure 4: “Category A”). The U.S. also shows this spectrum of behavior but in comparison to LMICs, in the U.S. many more originators have been replaced by their counterpart generic versions (Figure 4). This same question of switching from originator to a counterpart generic medicine was studied in 10 European countries between 2002 and 2006 [23]. Briefly, for countries that have long promoted generics such as Germany, the UK and the Netherlands there was an increase in the volume consumption of generic medicines and a switch from an original to its counterpart generic version. For less mature markets, such as Spain, Italy, Belgium and Austria, they found only an increase in generic medicines consumption with no ‘switching’. The same could be true for the LMIC studied; in markets such as Brazil and South Africa we found a higher number of originators which were replaced by their counterpart generic products.

In our view, increases in private sector LMIC generic market share for the medicines under study are not predominantly a response to patent expiries. Certainly in Brazil, we know that the top 30 molecules with the highest decrease in the originator group were off-patent so that the increase in generics by volume (Table 2) cannot be attributed to the ‘release’ of generics onto the market post-patent. In other countries (Colombia, Ecuador, Philippines, Venezuela, South Africa), very few “top 30” originators (i.e., orlistat, cyproheptadine, cefaclor, trimetazidine, glibenclamide, loratidine; citalopram, meloxicam, omeprazole, simvastatin) had both diagnostic ratios >1 (indicating complete replacement by the generic) and no generic counterpart at the 2000/2001 boundary. We can certainly infer from this a rapid generic replacement of the originator. We are less certain that this is a possible “signature” of patent expiry in-country sometime during 2001–2011 as we can neither confirm nor deny the patent expiration dates for these medicines.

It is thus tempting to assert that increases in generics in the LAC region over time (Figure 3, Table 1) and the majority of diagnostic values >0 (categories A and B: (Figure 4)), result from comprehensive policies, at least in Latin America, to promote substitution of originators with counterpart generic medicines [24] and not from patent expiration.

There are alternative explanations for the increase in generic medicines in the LAC region. One is the relative importance of generic substitution within pharmacies, another is the direct demand for generic medicines by consumers who buy medicines without prescription. However, our data does not permit us to clearly distinguish these alternatives . The literature suggests that generic substitution in pharmacies in some Latin America countries is prohibited if the brand name of the product is mentioned on the prescription (e.g. Mexico) [25]) and for some countries policies to promote INN prescribing has not resulted in a very significant uptake as they have not been enforced [26]. This suggests that the uptake of generic medicines in the private market may be more consumer-driven rather than driven by effective generic substitution policies. Thus, what may be driving the originator/generic dynamic is balance between a change to less costly options for consumers and a more profitable medicine because of better markups and rebates.

Another explanation are safety and efficacy concerns that are possibly responsible for some of the observed market dynamics. Originators losing market share without a corresponding generic market (“Category D” Figure 4; Table 3) include medicines already taken off the market in the United States (cox-2 inhibitors like rofecoxib). The reason for this behavior in other classes (e.g., alkylated antihistamines like diphenhydramine – sold as Benadryl® in the U.S. and halogenated derivatives e.g., chlorphenamine) may be due to removal of the originator from the market and/or substitution of another, more effective originator or even a substitutable, non-counterpart generic. We cannot at present distinguish between any of these possibilities.

Finally, as the population of a country ages and more non-communicable diseases are treated with medications, the consumer demand as well as demand by insurance schemes for less expensive and/or more cost-effective drug therapy has continued to grow. Elements driving the observed increase in generics in the LAC region, indeed in any country, are likely to be multifactorial.

### Limitations

A possible limitation is that we cannot capture the entire pharmaceutical market (private and public) of a given country so that we are not attempting to generalize our findings to the entire pharmaceutical market of each of the 19 LMIC. It the same time it is worthwhile mentioning that the private sector in LMICs overall is often more than 60% of the total medicines market by value [26]. In the specific countries that are the subject of this analysis, the percent of the total pharmaceutical market by value allocated to the private sector is even higher, averaging 76 % (median: 80%) [IMS unpublished data] so we are capturing a clear majority of the total medicines market.

We eliminated the “patent N/A” category from our analysis as they cannot be placed into any IMS category (i.e., “INN”, “originator”, “branded generic”). This category averages across all countries about 12% of the total private market (including patent N/A). Thus, we are still capturing a substantial part of the total private sector pharmaceutical market in the 19 LMICs.

However, we are analyzing a limited number of countries for each region, aside from Latin America. Thus, we cannot really generalize the data geographically to Asia or the MeSA as a whole. Nonetheless, some of the largest sized pharmaceutical markets in each of the regions are included (e.g. Egypt, in the MeSA and the Philippines in Asia).

We assume there are systematic errors in our panel data. Any systematic errors in the panel data are due to several factors: (1) Coverage: Some distribution channels are not captured in our data and therefore, not included in the analysis. Our study focuses on the private market and excludes the public sector. (2) Accuracy: Accuracy may vary by product size for sample-based data, as most audits are sample based. A more cogent limitation might be the fact that there are almost inevitable errors in the panel data due to changes in already existing distributors over time. We attempted to model the impact of changes in volume on outcome measurements which should be the most sensitive to such changes. We infer that the errors in outcome measures are rather small. We do not think that changes in market share that we see over time are caused by changes in the number of auditing entities included in annual surveys. Unknown and/or uncorrected under/overestimations would have to occur continuously over multiple years in order to account for the trends we observe. This seems unlikely.

DDD is the more commonly used measure of medicines consumption in the scientific literature. However, as we represent the values as ratios, it is unlikely that analysis by DDD would produce different results.

Clearly differentiating between products that were off-patent from the beginning of the study period and those that lost their patent during the study period would add an additional insight into our results regarding the increase in generic medicines market share. We note that patent protection has not always been enforceable, or enforced, in all countries. This is a limitation of the study. However, for all the “top 30” originators in each country under study, we were able to determine whether there were any generic competitors on the market prior to the beginning of the time series. Changes in generic market share thus occurred in some of the cases even in the presence of originator and within, not prior, to the study period.

Lastly, any inferences we draw regarding patent expiry should have been obtained from the respective LMIC Patent Offices but we did not have this information and this is almost always difficult in any case for LMICs [27].

## Conclusion: Future Challenges and Policy Implications

There are few private retail sector analyses of generic medicines in LMICs. Our study shows that generic medicines (branded generic plus INN generic) represent between 70 and 80% of market share in the private sector of these LMICs which is greater than most European countries. In contrast to high income markets such as the U.S., branded generic medicine market share is much higher than unbranded generics, most notably in countries in the MeSA and Asia. Although switching from originator to generic counterparts saves money in principle [11], this narrative in reality is complex and nuanced at the level of individual medicines.

Our study is a first step analyzing generic medicines consumption in the private market in LMIC over time. For some countries with an originator medicines market share of around 30% we found less than 3% change in the generic medicines market share over time (e.g. Argentina, Pakistan, Egypt and Tunisia). For various reasons, generics may not be promoted, but the conditions under which we can say that generics would be taking more market share in these countries are not known with certainty. It is possible that there is a lack of effective policies promoting generic medicines. For other countries it is difficult to say from this analysis what is actually driving the decreasing market share of originators in, for instance, many Latin American countries, South Africa and the Philippines. Generic medicine policies such as specific pricing policies, aligning financial incentives of consumers and prescribers/dispensers, promoting generic medicines among consumers, economic forces (e.g., presence/absence of taxes, rebates, discounts), safety recalls and health care provision (e.g., presence/absence of health insurance coverage, presence of fragmented and complex distribution channels) might play a role.

A second important step would be a more rigorous and in-depth economic and policy analyses to establish cause-effect relationships between pharmaceutical policies and, for example, the data presented here. Those studies can support relevant recommendations on medicines policies and assist in modulating their implementation in-country. A comprehensive prospective picture that includes estimations of the number of generic competitors, penetration of generics after patent expiry, and national-level costs of purchasing branded versus unbranded generics will require accurate, validated price information as well as a well-described policies and their implementation process. In addition, the analysis should be complemented by a qualitative review of policy changes and their likely effect on the volume share. Interviews with policy makers, policy analysts and other stakeholders can provide valuable insight into the market dynamics.

## Supporting Information

Annex S1 (XLSX)
